# Illustrations of Hospital Practice

**Published:** 1860-10

**Authors:** 


### ILLUSTRATIONS OF HOSPITAL PRACTICE.

### PENNSYLVANIA HOSPITAL.—SERVICE OF DR. MEIGS.

*Herpes Zoster.*—A strong, robust sailor came into the hospital on Monday last, having been sick for five days. He had first been taken with a pain in his right side, of a sharp, extremely severe character. He complained then of nothing else but this pain. Three days later an eruption appeared on the back of his right side, and extending around the loin, on that side. It presented the well marked characteristics of herpes zoster. The pain still continues, but not as severe as before the eruption.

Herpetic eruptions are generally associated with a disordered state of the blood. The pain is characteristic of the disease ; it frequently continues for weeks, and sometimes even months after the eruption has gone.

The treatment in this case was a blue pill at night, followed by castor oil. Locally a belladonna plaster may be applied, or a liniment, composed of tincture of opium, aconite, and sweet oil. Watson recommends to give iodide of potassium and quinine, if the pain is very severe.

*Pathological Specimens—Remarks.*—The patient from whom the first specimens were obtained had entered the hospital on the morning of ——. He was thirty years of age, and for some time back had been very dissipated, having had several attacks of delirium tremens. He presented the symptoms of approaching delirium tremens when entering; white tongue, tremulous movements, pulse somewhat frequent, pale countenance; he was put on tincture of bark and lupulin. He did not sleep. Toward evening on the day of entrance his pulse rose to 120, and still later at night he became suddenly worse. The pulse rose to 130-40; the patient sank in a comatose condition, and died at four o'clock in the morning.

*On Post-mortem examination*, a very small amount of urine was found in the bladder, and this, on being tested in the ordinary method, was found to contain albumen. This strengthened the suspicion that his death was due to uræmic poisoning, rather than to delirium tremens.

The *liver* was somewhat enlarged, and on microscopical examination, found to be loaded with fat cells, and oil globules.

The *kidneys* presented the usual appearances of atrophy from granular degeneration with the microscope, and were found to be deprived of their epitheium, and the cortical substances very much diminished.

The *heart* was not fatty; the *lungs* were healthy.

Uræmia in this case was the chief cause of death. As the renal degeneration advances in these cases, and the kidneys fail to eliminate the urea from the blood properly, the urea gradually accumulates in the system, and, acting upon the nerve centers, as an irritant narcotic poison, produces often, very suddenly, convulsions, delirium, or as in this case, coma and death.

*Diphtheria.*—The next specimens presented were taken from a man 24 years of age, a strong, well built German, tailor by trade, who came to the hospital on Thursday afternoon, at 4 o'clock, P. M.

He stated, on his admission, that he had been taken sick on Saturday afternoon previously, but did not feel very sick until Thursday, when he walked to the hospital, a considerable distance.

His pulse was then 122; all the faucal structures were much tumified; the palatine arch, tonsils, uvula covered with a dirty white, greyish exudation; the external cervical lymphatic glands behind and below the ramus of the lower jaw were tumified and hard. There was, and this is of especial importance in acute diseases of the fauces, in croup, scarlatina anginosa, maligna, diphtheria, etc., acute œdema below the chin; there was difficulty of swallowing, his breathing labored, more frequent than natural; there was stridor trachealis, not, however, the croupy stridor. In adults we rarely find, even in true membraneous croup, the confirmed croupy stridor of infancy, on account of the different anatomical relations of the tracheal and laryngeal passages. Articulation was indistinct.

The patient became rapidly worse; at night the pulse was 132; he sank rapidly, and died toward morning. He was conscious almost to the last.

A *post-mortem* examination was made, and the specimens are here presented.

You see the tongue, the palatine arch, tonsils, uvula, all swollen, and covered, more or less, with patches of exudation of a dirty, greyish color; going into the trachea we find a thick peseudo-membrane filling it almost completely, and extending into the secondary and tartiary ramifications.

Within a very short time some continental writers have stated in journals, that *albumen is almost always present in diphtheria.* We have examined the urine of this patient, and here you see quite a copious precipitate of albumen thrown down on the application of the heat and nitric acid tests. This is the first case in which I have had occasion to observe this phenomenon.

There is no doubt, from this and other facts, connected with this disease, that it depends upon a blood poison. Some believe it to be identified with, or amalgous to scarlet fever, depending upon the same cause. Yet, though both diseases may have something alike, both being blood diseases, yet we cannot consider them the same. Second attacks of scarlet fever are very rare. A patient who has had scarlet fever once, is almost sure to be protected from another attack of the same disease. Not so with diphtheria. Diphtheria does not protect against scarlet fever, nor *vice versa.*

In reference to the treatment of this, generally mild, but not unfrequently formidable disease, I prefer in the early stages the golden sulphate of antimony, in combination with Dover's powder;—say $\frac{1}{4}$ of a grain of the former to $\frac{1}{2}$ a grain of the latter. Absolute repose in bed is imperatively demanded, as in all diseases depending upon a blood-poison. In the latter stages, and when the disease is of a very adynamic type, iron and quinine are indicated.

As a local application I prefer capsicum. The patient may either drink capsicum tea, or his throat may be swabbed with a strong infusion of capsicum.

A good formula for the latter purpose is,

℞. Capsici., ʒ ii.

Acid. gallic., ℈ ii.

Aquæ, f ℥ i to f ℥ iss.

Stimulating applications around the throat and neck are of service. A very good one is a mixture of equal parts of tincture of capsicum and tincture of cantharides, to be applied until the skin is reddened, and repeating when the effect begins to pass off.—*Med. and Sur. Reporter*, *Sept.*, 1860.

